# Relationships between Loneliness and Occupational Dysfunction in Community-Dwelling Older Adults: A Cross-Sectional Study

**DOI:** 10.1155/2023/9505865

**Published:** 2023-09-07

**Authors:** Daiki Nakashima, Keisuke Fujii, Yuta Kubo, Kyosuke Yorozuya

**Affiliations:** ^1^Department of Rehabilitation, Faculty of Health Science, Naragakuen University, 3-15-1, Nakatomigaoka, Nara, Nara 631-8524, Japan; ^2^Department of Rehabilitation Occupational Therapy Course, Faculty of Health Science, Suzuka University of Medical Science, 1001-1, Kishioka, Suzuka, Mie 510-0293, Japan; ^3^Division of Occupational Therapy, Faculty of Rehabilitation and Care, Seijoh University, 2-172 Fukinodai, Tokai, Aichi 476-8588, Japan

## Abstract

The study explored cross-sectional associations between loneliness and occupational dysfunction in community-dwelling older adults. Seventy-four older adults (12 men and 62 women; mean age 73.9 ± 8.3 years) completed a questionnaire survey that included the Japanese version of the UCLA Loneliness Scale Version 3 and the Classification and Assessment of Occupational Dysfunction (CAOD). Bayesian statistical modeling was used for a more stable estimation given the small sample. For model selection, we assumed a univariate analysis model of the CAOD (Model 1); a multivariate analysis model, including confounding factors in Model 1 (Model 2); and a multivariate analysis model, including random effects in Model 2 (Model 3). The best model was selected by comparing the widely applicable information criterion (WAIC) and the widely applicable Bayesian information criterion (WBIC) for each model. Bayesian statistics with the dependent variable as “loneliness” showed that the best model used “occupational dysfunction” as the independent variable and included confounding factors and random effects (WAIC = 474.5 and WBIC = 213.1). The best model identified an association between occupational dysfunction and loneliness (odds ratio [OR] = 2.363; 95% Bayesian confidence interval [CI] = 1.105–5.259). This study highlights the role of occupational dysfunction in addition to the risks and related factors reported to date when dealing with loneliness. Therapists could help older adults cope with loneliness by supporting their social participation and improving their occupational dysfunction.

## 1. Introduction

The population of older adults is increasing globally as the birth rate declines and life expectancy increases; it is estimated to reach 15.9% of the world population by 2050 [1]. The aging rate in Japan is high, and many older adults experience individual and social changes such as deteriorating economic conditions, a decline in physical function, and bereavement [2]. In recent years, the loneliness associated with such individual and social changes has been cited as an important social issue [3], especially problematic in old age, where many losses are experienced [2]. The number of people who experience loneliness is increasing each year [4], and a 2018 survey showed the proportion of lonely people to be 20% in the United States and the United Kingdom and 10% in Japan [3].

Loneliness is defined as a distressing feeling that accompanies the perception of inadequate quantity or quality of one's social relationships [5] and can be explained as the discrepancy between the relationships one desires and those one has [6]. Loneliness is known to cause physical and psychological health hazards such as decreased physical activity [7], decreased activities of daily living (ADLs) [6], and increased depressive symptoms [8]. In recent years, it has become clear that loneliness leads to a higher mortality rate than smoking and lack of physical activity [9]. Therefore, it is urgent to examine methods that can reduce loneliness when considering disease prevention and health promotion in older adults.

In occupational therapy, few studies have focused on loneliness [10–12]. No consensus exists on the effectiveness of interventions such as community-based group rehabilitation and community mentoring by occupational therapists. Therefore, we focused on occupational dysfunction as a new perspective. However, research on occupational dysfunction and loneliness remains insufficient. Occupational therapists provide evaluation, treatment, and support focusing on client dysfunction. Occupation denotes various activities such as work, business, leisure activities (play), ADLs, and social participation [13]. Occupational dysfunction is a negative experience associated with these daily activities and comprises four elements: occupational deprivation, occupational alienation, occupational imbalance, and occupational marginalization [13]. Occupational deprivation is a condition in which there is a lack of opportunity for daily activities due to external factors such as “There is no place to enjoy hobbies,” and “I don't have the opportunity to perform occupations that are important to me” [14, 15]. Occupational alienation is a condition in which meaning cannot be found in daily activities, such as “I feel that my life is meaningless” and “There is no sense of accomplishment in daily activities” [14, 15]. Occupational imbalance is a condition in which the balance of daily activities is confused, such as “My daily rhythm is disturbed because I'm so busy” [14, 15]. Occupational marginalization is a condition in which meaningful daily activities are not recognized by the surroundings, such as “I have opinions, but nobody approves of them” [14, 15]. Occupational dysfunction can occur not only in persons with disabilities but also in healthy persons [15]; in a study investigating social isolation and occupational dysfunction in community-dwelling older adults, about 15% of the participants demonstrated occupational dysfunction [14]. Occupational dysfunction has become a significant problem, harmful to human health [16]. In the literature, risk factors leading to loneliness are living alone, ADL impairment, being female, lower income, lower education, subjective causes (i.e., illness, death, and lack of friends), poor self-reported health, depression, lack of meaning in life, being poorly understood by others, and environmental factors [17–19]. However, they are also related to occupational dysfunction [15, 20]. Therefore, we hypothesized that older adults with occupational dysfunction could experience loneliness. This study examined the cross-sectional relationship between occupational dysfunction and loneliness among community-dwelling older adults. Exploring the link between occupational dysfunction and loneliness will lead to measures to reduce loneliness, a global social problem. It will also show occupational therapy's role in this field.

## 2. Materials and Methods

### 2.1. Materials

The study area was Nara City, Nara Prefecture, Japan. Nara is a city with a population of 350,767, located in the central part of Japan, with an aging rate of 31.1% [21]. In this study, 90 older adults aged 65 and older, who participated in a general long-term care prevention project from August to October 2019, were included. The following exclusion criteria were applied: (1) those who had been certified as needing support or long-term care and (2) those who could not complete the questionnaire. This study used a cross-sectional design.

### 2.2. Ethical Considerations

This study was conducted after obtaining approval from the ethics review committee of the authors' affiliated organization. The outline of the study was then explained to the participants verbally and on paper, and their consent was obtained (approval number: 31-022).

### 2.3. Measurement Variables

We asked for answers to the following items using the questionnaire survey method: age, gender, household composition (Do you live alone? Yes or no), education (junior school graduate, junior high school graduate, high school graduate, or college graduate), economic condition (very difficult, slightly difficult, normal, somewhat rich, or very rich), depression, the Japan Science and Technology Agency Index of Competence (JST-IC), the Japanese version of the abbreviated Lubben Social Network Scale (LSNS-6), the Japanese version of the UCLA Loneliness Scale Version 3 (UCLA-LS3-J), and the Classification and Assessment of Occupational Dysfunction (CAOD).

#### 2.3.1. Depression

Depressive status was assessed using the depression items from the Kihon Checklist [22]. The Kihon Checklist is a scale created by the Japanese Ministry of Health, Labour and Welfare to measure frailty in older adults. It consists of 25 items related to instrumental ADLs (3 questions), social ADLs (4 questions), physical functions (5 questions), nutritional status (2 questions), oral function (3 questions), cognitive function (3 questions), and depression (5 questions) [23]. Response options constitute yes or no for each item, and the score ranges from 0 (no frailty) to 25 (high frailty) [22]. In this study, depression was defined as answering yes to two or more of the five depression items [23, 24]. Higher values indicate poorer mental health (range: 0–5 points). The validity of the Kihon Checklist has been verified through surveys of older adults [23].

#### 2.3.2. The Japan Science and Technology Agency Index of Competence

The JST-IC is a scale for evaluating higher-level living functions of older adults and is composed of 16 items: technology usage (4 questions), information practice (4 questions), life management (4 questions), and social engagement (4 questions). Higher JST-IC scores indicate better living function [25]. The validity of the JST-IC has been verified through surveys of older adults [25].

#### 2.3.3. The Japanese Version of the Abbreviated Lubben Social Network Scale

The LSNS-6 is a measure of social networks in older adults. The scale consists of six items: three items relating to family networks (How many relatives do you see or hear from at least once a month? How many relatives do you feel at ease with that you can talk about private matters? How many relatives do you feel close to such that you could call on them for help?) and three items relating to friend networks (three items in which the word “relatives” in these questions is replaced with “friends”). For each item, we requested responses about the number of people in the network using six grades from 0 (none) to 5 (nine or more). The score ranges from 0 to 30 points, and the higher the score, the more abundant the social networks are judged to be [26]. The validity and reliability of the LSNS-6 have been verified through surveys of older adults [26].

#### 2.3.4. The Japanese Version of the UCLA Loneliness Scale Version 3

The UCLA-LS3-J is a measure of loneliness and consists of 20 items. For each item, answers are requested using four grades of 1 (*never*), 2 (*rarely*), 3 (*sometimes*), and 4 (*always*), and the score ranges from 20 to 80 points. The UCLA-LS3-J shows that the higher the score, the higher the feeling of loneliness [27]. The validity and reliability of the UCLA-LS3-J have been verified through surveys of mothers with infants, toddlers, and older adults [27, 28]. Cronbach's alpha of the UCLA-LS3-J in the previous study was 0.926 [27], and in this study, it was 0.888.

#### 2.3.5. Classification and Assessment of Occupational Dysfunction

The CAOD is a 16-item scale for assessing occupational dysfunction and includes four factors: occupational deprivation (3 questions), occupational alienation (3 questions), occupational imbalance (4 questions), and occupational marginalization (6 questions) [13]. For each item, answers were requested using seven grades from 1 (*not applicable*) to 7 (*applicable*), and the score ranged from 16 to 112 points. The cut-off value of the CAOD was 52 points; the higher the score, the more the occupational dysfunction. The validity and reliability of the CAOD have been verified in surveys of university students and medical professionals [13, 20]. Cronbach's alpha of the CAOD in the previous study was 0.914 [13], and in this study, it was 0.921.

### 2.4. Statistical Analysis

This study employed Bayesian statistical modeling to make a more stable estimation with a small sample size. In Bayesian statistics, a parameter distribution is generated as a posterior distribution from the obtained data by using the prior distribution and combining Bayesian estimation with the Markov chain Monte Carlo method, which is a random number generation algorithm. As it does not depend on a theory that requires a large sample size, stable analysis is possible even for data with a small number of samples, and it was judged to be suitable for this study [29, 30].

As the UCLA-LS3-J score as the dependent variable is a discrete variable and this data may be overdispersed, a model assuming a hierarchical structure was considered. Therefore, a binomial logistic regression model with random intercepts of participants (individual differences) was used as the statistical model. The parameters were estimated using the Markov chain Monte Carlo method, and the half-Cauchy distribution recommended as a weakly informative prior distribution was used for the prior distribution [29]. The settings were as follows: chains 4, iteration 2000, warm-up 1250, and thin 1. The appropriateness of the estimated posterior distribution was judged to have converged when Rhat was less than 1.05 [30].

Age, gender, education, economic conditions, household composition, and depression items in the Kihon Checklist, JST-IC, and LSNS-6 were chosen as independent variables and were selected from previous studies [17–19]. For model selection, we assumed a univariate analysis model of the CAOD (Model 1), a multivariate analysis model including confounding factors in Model 1 (Model 2), and a multivariate analysis model including random effects in Model 2 (Model 3). The best model was selected by comparing the widely applicable information criterion (WAIC) and the widely applicable Bayesian information criterion (WBIC) for each model (half-Cauchy prior and binomial distribution with random effects for individual differences) [31]. The robustness of the model was compared with the model in which the prior distribution (noninformative prior distribution) and the data distribution (normal distribution) were changed with respect to the model judged to be the best, and it was confirmed that the results did not change significantly. In addition, the relative strength of influence of the two models (null hypothesis model [Model 0], covariates, and mixed models excluding only the CAOD [Model 4]) and the model judged to be the best were calculated using the Bayes Factor (BF). BF is an index that determines which hypothesis is supported by the ratio of the likelihoods of the two hypothesis models (difference in log-likelihood) and is similar to a hypothesis test in conventional statistics [32]. The difference from conventional statistics is that the null hypothesis may be supported because BF calculates the plausibility of both hypotheses. In this study, we adopted the logarithmic BF (log_10_(BF_*ij*_)) criterion as the size of evidence for the null hypothesis by Jeffreys [33]: 0 to 0.5 (not worth more than a bare mention), 0.5 to 1 (substantial), 1 to 2 (strong), and >2 (decisive) [32, 34].

Bayesian statistical modeling confirmed the results by the posterior median and 95% Bayesian confidence interval (CI). The 95% Bayesian CI in Bayesian statistics has a similar meaning to the 95% Bayesian CI in conventional statistics and is interpreted as significant if the value does not contain zero (an interpretation that does not rely on the *P* value is possible). In this study, odds ratios (OR) were calculated from the sampled estimation results, and the posterior median and 95% Bayesian CI were used as the results. Statistical software R (version 4.0.5; R Foundation for Statistical Computing, Vienna, Austria) with the RStan (version 2.21.2) package, loo (version 2.4.1) package, and bridge sampling (version 1.1-2) packages were used for statistical analysis.

## 3. Results

### 3.1. Completed Questionnaires

**Figure 1:**
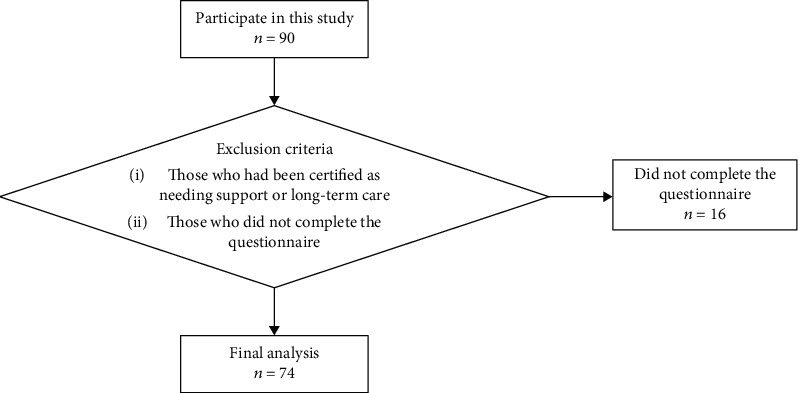
Flowchart for participant recruitment.

We excluded 16 participants, who did not complete the questionnaires of the measurement variables used in the statistical analysis. None of the participants was certified as requiring support or long-term care. Finally, 74 participants were included in the analysis (Figure 1).

### 3.2. Model Robustness

In Bayesian statistical modeling, all models were estimated as predicted distributions that approximated the true distribution (Rhat < 1.05). In Model 1, WAIC = 738.8 and WBIC = 365.0; in Model 2, WAIC = 667.4 and WBIC = 316.6; in Model 3, WAIC = 474.5 and WBIC = 213.1; and Model 3 was selected as the best model. Regarding the robustness of the model, the results did not change even with the model in which the prior distribution and data distribution were changed in Model 3, and it was judged to be robust. In addition, as a result of calculating the BF of Model 3, the log_10_(BF_30_) of the null hypothesis model (Model 0) was 118.248, thus showing that Model 3 was definitely recommended as the best among the assumed models in this study. Furthermore, log_10_(BF_34_) comparing Model 3 and Model 4 was 2.502, thereby demonstrating the strong usefulness of Model 3, including CAOD. The Bayesian statistical modeling of the CAOD components performed as a secondary analysis was also generated as a predicted distribution that approximated the true distribution (Rhat < 1.05), and the model was also judged to be robust.

### 3.3. Participants' Characteristics

Table 1 presents the characteristics of the participants. The average age of the 74 participants for the final analysis of this study was 73.9 ± 8.3 years, and 83.8% were female. About 85% of the respondents answered that they had 12 years or more of education, and about 80% stated that their economic situation was normal or above average.

### 3.4. Variables Associated with Loneliness

Table 2 presents the relationship between occupational dysfunction and loneliness. The Bayesian statistics confirmed the association between occupational dysfunction and loneliness, even after adjusting for confounding factors, including random effects (OR = 2.363; 95%Bayesian CI = 1.105–5.259).

## 4. Discussion

To the best of our knowledge, this study is the first to investigate the relationship between occupational dysfunction and loneliness. Fujii et al. [14] investigated the association between occupational dysfunction and social isolation and reported that occupational dysfunction affects social isolation, even after adjusting for confounding factors [14]. This study focused on loneliness, which indicates the subjective state of social relations—unlike social isolation, which indicates the objective state of social relations, such as the number and frequency of social connections. Loneliness and social isolation can independently affect people's health, and public health should consider these conditions separately [19]. An analysis using the Bayesian statistical model revealed that occupational dysfunction was associated with loneliness. Occupational dysfunction has been reported to be associated with depression [20], while depression has also been associated with loneliness [17]. Although this study was not able to perform an analysis that considers the health-related quality of life, occupational dysfunction and loneliness have a somewhat independent relationship because the analysis results consider depression and social networks. For example, a state of occupational dysfunction is a negative experience in daily life, that is, being in a state of “not being accepted for my work/opinion” and “being denied by others what I like to do.” These conditions also include pessimistic feelings felt through others. Therefore, occupational dysfunction is considered to be associated with loneliness manifesting negative emotions in social relationships. In addition to the results of this study, as people with occupational dysfunction in previous studies were socially isolated, occupational dysfunction may affect the quality and quantity of social relationships and how they feel about them. Therefore, it is important to have a perspective on occupational dysfunction in addition to the risks and related factors that have been reported thus far when dealing with loneliness. Previous studies have reported the need to identify treatment elements for loneliness and the optimal frequency and duration of such treatment [35]. In this cross-sectional survey, it would be challenging to determine the causality of the results. However, a new perspective on loneliness has been proposed in this study.

The occupational dysfunction as measured by the CAOD comprises the components of occupational deprivation, occupational alienation, occupational imbalance, and occupational marginalization. Occupational deprivation may be affected by external problems. As the effects of environmental factors such as environmental barriers and inadequate resources for socializing have been reported as risk factors for loneliness [18], occupational dysfunction, including elements of occupational deprivation and loneliness, is also supposedly related. For example, if daily activities are obstructed by external factors such as geographical problems, one may face challenges that cannot be dealt with alone. In other words, the inability to build the social relationships that one seeks due to external factors can lead to loneliness. Loneliness is related to a lack of meaning in life and being poorly understood by others [17, 19]. Therefore, occupational alienation and occupational marginalization are also considered to affect loneliness. A study investigating the relationship between occupational dysfunction and social isolation reported that the odds ratio for social isolation was low in the case of an occupational imbalance [14]. It shows that the number of social relations can be maintained even when daily life is busy. A person may feel lonelier when they are socially connected but busy, and the quality of communication is not what is desired.

### 4.1. Limitations

This study is the first to analyze the association between occupational dysfunction and loneliness, but it has some limitations. First, as it was a cross-sectional study, the causal relationship between occupational dysfunction and loneliness was not clarified. Therefore, longitudinal studies are needed to investigate whether occupational dysfunction is a risk factor for loneliness. Second, the sample size was small. To address this problem, we performed an analysis using Bayesian statistical modeling. However, Bayesian statistics are affected by the data characteristics observed and may not reflect the characteristics of the population in other areas. Therefore, in the future, it is necessary to conduct the same survey targeting different regions and update the results (Bayesian update) using this data as a prior distribution. Third, there was a large difference in the gender distribution of the participants; the proportion of women was high. The analysis was controlled for gender in this study. However, future consideration of gender will help examine support that accounts for the target population's characteristics. Fourth, as the survey method used was a self-report questionnaire, the participants may have overestimated or underestimated their responses. Fifth, as the study sample comprised participants in a general long-term care prevention project, it is possible that they were a group with high health consciousness.

## 5. Conclusion

This study used a Bayesian statistical model to clarify the relationship between occupational dysfunction and loneliness. Although some research limitations exist, the results of this study suggest a role for occupational therapy and a new perspective on loneliness. The occupational therapists may be able to help older adults cope with loneliness by supporting their occupational participation and improving their occupational dysfunction. Future studies will be required to ascertain whether occupational therapy practices can improve loneliness.

## Figures and Tables

**Table 1:** Participants' characteristics.

Characteristic	Frequency (*n* = 74)
Age, mean ± SD (years)	73.9 ± 8.3
Female (%)	83.8
Education (%)	Junior school graduate: 0 Junior high school graduate: 13.7 High school graduate: 47.2 College graduate: 39.1
Economic conditions (%)	Very rich: 1.4 Somewhat rich: 16.2 Normal: 62.1 Slightly difficult: 16.2 Very difficult: 4.1
Living alone (%)	32.4
Depression (score), medians (first-third quartile value)	0 (0–1)
JST-IC (score), medians (first-third quartile value)	13.5 (11–15)
LSNS-6 (score), medians (first-third quartile value)	17.5 (15–20)
Occupational dysfunction (%)	23.0
CAOD (score), mean ± SD	38.9 ± 18.0
UCLA-LS3-J (score), mean ± SD	39.3 ± 10.7

SD: standard deviation; JST-IC: Japan Science and Technology Agency Index of Competence; LSNS-6: Japanese version of the abbreviated Lubben Social Network Scale; CAOD: Classification and Assessment of Occupational Dysfunction; UCLA-LS3-J: Japanese version of the UCLA Loneliness Scale Version 3.

**Table 2:** Relationship between occupational dysfunction (CAOD total score) and loneliness.

	Model 1^∗^		Model 2^†^		Model 3^‡^	
	OR	95% Bayesian CI	OR	95% Bayesian CI	OR	95% Bayesian CI
CAOD	3.353	2.535–4.437	2.271	1.649–3.127	2.363	1.105–5.259

Binomial logistic regression with random intercepts for subjects using the Markov chain Monte Carlo approach was used. OR: odds ratio; CI: confidence interval; CAOD: Classification and Assessment of Occupational Dysfunction. ^∗^Model 1 is a model with no adjustment. ^†^Model 2 had no random effects, but adjusted for age, gender, education, economic conditions, household composition, depression, instrumental activities of daily living, and social isolation. ^‡^Model 3 had a random effect, adjusting for age, gender, education, economic conditions, household composition, depression, instrumental activities of daily living, and social isolation.

## Data Availability

The data supporting this study's findings are available on request from the corresponding author. The data are not publicly available due to privacy or ethical restrictions.

## Supplementary Materials

Supplementary Materials The supplementary file is the Statement on Enhancing Observational Study Reporting in Epidemiology.
